# The PID Odyssey 2030: outlooks, unmet needs, hurdles, and opportunities — proceedings from the IPOPI global multi-stakeholders’ summit (June 2022)

**DOI:** 10.3389/fimmu.2023.1245718

**Published:** 2023-08-15

**Authors:** Susan Tadros, Johan Prévot, Isabelle Meyts, Silvia Sánchez-Ramón, Nahla H. Erwa, Alain Fischer, Guillaume Lefevre, Matthew Hotchko, Peter M. Jaworski, Helen Leavis, Cornelis Boersma, Jose Drabwell, Martin van Hagen, Samya Van Coillie, Martine Pergent, Siobhan O. Burns, Nizar Mahlaoui

**Affiliations:** ^1^Department of Immunology, Royal Free London NHS Foundation Trust, London, United Kingdom; ^2^IPOPI, Brussels, Belgium; ^3^Department of Pediatrics, University Hospitals Leuven, KU Leuven, Leuven, Belgium; ^4^Department of Microbiology, Immunology and Transplantation, KU Leuven, Leuven, Belgium; ^5^Department of Immunology, Health Research Institute of the Hospital Clínico San Carlos (IdISSC), IML and IdISSC, Madrid, Spain; ^6^Faculty of Medicine, University of Khartoum, Khartoum, Sudan; ^7^Pediatric Hematology-Immunology and Rheumatology Unit, Necker-Enfants malades University Hospital, Assistance Publique-Hôpitaux de Paris (AP-HP), Paris, France; ^8^French National Reference Center for Primary Immune Deficiencies (CEREDIH), Necker-Enfants malades University Hospital, Assistance Publique-Hôpitaux de Paris (AP-HP), Paris, France; ^9^Collège de France, Paris, France; ^10^Imagine Institute, UMR Inserm 1163, Paris, France; ^11^Univ. Lille, Inserm, CHU Lille, U1286 – INFINITE Institut de recherche translationnelle sur l'inflammation, Lille, France; ^12^Institut d'Immunologie, CHU Lille, Lille, France; ^13^Marketing Research Bureau, Inc., Yakima, WA, United States; ^14^Strategy, Ethics, Economics, and Public Policy, McDonough School of Business, Georgetown University, Washington, DC, United States; ^15^Department of Rheumatology & Clinical Immunology, University Medical Center (UMC), Utrecht University, Utrecht, Netherlands; ^16^Health-Ecore B.V., Zeist, Netherlands; ^17^Unit of Global Health, Department of Health Sciences, University Medical Center Groningen (UMCG), University of Groningen, Groningen, Netherlands; ^18^Department of Management Sciences, Open University, Heerlen, Netherlands; ^19^Department of Internal Medicine, Division of Allergy & Clinical Immunology, Erasmus University Medical Center Rotterdam, Rotterdam, Netherlands; ^20^Department of Immunology, Erasmus University Medical Center Rotterdam, Rotterdam, Netherlands

**Keywords:** primary immunodeficiencies/inborn errors of immunity, immunoglobulin replacement therapy/plasma derived medicinal products, hematopoietic stem cell transplantation, gene therapy, newborn screening, next generation sequencing, quality of life, targeted therapies/personalized medicine

## Abstract

IPOPI held its first Global Multi-Stakeholders’ Summit on 23-24 June 2022 in Cascais, Portugal. This IPOPI initiative was designed to set the stage for a stimulating forward-thinking meeting and brainstorming discussion among stakeholders on the future priorities of the PID community. All participants were actively engaged in the entire Summit, bringing provocative questions to ensure a high level of discussion and engagement, and partnered in identifying the outlooks, unmet needs, hurdles and opportunities of PIDs for 2030. The topics that were covered include diagnosis (e.g., newborn screening [NBS], genomic sequencing— including ethical aspects on the application of genomics on NBS, the role of more accurate and timely diagnostics in impacting personalized management), treatment (e.g., the therapeutic evolution of immunoglobulins in a global environment, new therapies such as targeted therapies, new approaches in curative therapies), the interactions of Primary ID with Secondary ID, Autoinflammatory Diseases and other diseases as the field experiences an incessant evolution, and also the avenues for research in the field of humanities and human sciences such as Patient-Reported Outcome Measures (PROMs), Patient-Reported Experience Measures (PREMs), and Health-Related Quality Of Life (HRQoL). During this meeting, all participants contributed to the drafting of recommendations based on our common understanding of the future opportunities, challenges, and scenarios. As a collection of materials, perspectives and summaries, they are succinct and impactful and may help determine some of the next key steps for the PID community.

## Introduction

In recent years, the International Patient Organisation for Primary Immunodeficiencies (IPOPI) has been working with national primary immunodeficiency (PID) organisations and collaborating with scientific and medical institutions and experts, to bring the principles of care of PIDs closer to individuals living with these conditions (1, 2). With the view to continue and expand this effort, IPOPI held its first Global Multi-Stakeholders’ Summit on 23-24 June 2022 in Cascais, Portugal. This IPOPI initiative was designed to set the stage for a stimulating forward-thinking meeting and brainstorming discussion among stakeholders on the future priorities of the PID community. The uniqueness of this Summit is to bring together, in a working format designed to provoke a prospective and multi-dimensional discussion, a broad range of PID stakeholders: physicians (both pediatricians and adult physicians), scientists, clinician-scientists, ethicists, specialists in health-economics, patients and patient representatives coming from several continents.

All participants were actively engaged in the entire Summit, bringing provocative questions to ensure a high level of discussion and engagement addressing a selection of themes. For this very first edition, the key topics were: (i) the therapeutic evolution of immunoglobulins, (ii) personalized management of PIDs and (iii) the known and unknown facets of a constantly evolving PID field/what will the future bring?

Participants partnered in identifying the outlooks, unmet needs, hurdles, and opportunities of PIDs for 2030. This proceedings’ paper briefly reviews where the field of immunodeficiency currently stands in terms of access and supply of immunoglobulins, diagnostics and treatment, and the assessment of quality of life. In addition, it summarizes where we should aim to be in the next 10-20 years in these areas and what practical steps are required to ensure these goals are achieved. The ultimate goal of the Summit and this proceedings paper is therefore to help determine some of the next key steps for the PID community.

## Part 1: the therapeutic evolution of immunoglobulins: alternatives, access and supply

Immunoglobulin (Ig) replacement therapy (IgRT) remains a crucial part in the management of patients with primary and secondary immunodeficiencies since it was initially used in 1952 in agammaglobulinemic patients suffering from lethal recurrent pneumococcal invasive infections (3). The indications for IgRT have expanded and may vary depending on region (4, 5), with PID and secondary immunodeficiency (SID) still being major indications for treatment (6, 7).

IgRT is now administered *via* different routes including intravenous immunoglobulin (IVIg), subcutaneous immunoglobulin (SCIg) — either self-administered *via* pump or push methods — and facilitated SCIg, providing patients and clinicians with increased treatment options (8, 9). For PID patients, it is crucial to ensure that a range of IgRT options is available in order to enable optimal personalized care, as individual patient needs and product tolerability differ. In a large group of patients with antibody deficiencies, the incidence of recurrent infections and bronchiectasis despite receiving properly administered IgRT may be a challenge. Advances in IgA and IgM enriched immunoglobulins are taking shape and clinical trials to assess whether patients are at a greater risk to develop recurrent and/or chronic airways infections are ongoing.

Over the last 25 years, the global plasma protein market has expanded with demand for Ig steadily increasing at an annual growth rate of 7.4% per year for a broad range of indications (10). The increase in demand for Ig has been driven by multiple factors including increasing awareness and improved diagnosis of PID and expanding numbers of patients with SID. Also, there is an increase in the number of patients with autoimmune conditions (such as neurological indications — e.g., chronic inflammatory demyelinating polyradiculoneuropathy (CIDP) and myasthenia gravis (MG) — which typically require a much higher quantity of Ig preparations as patients with PID or SID (2 g/kg per month vs 0.4-0.6 g/kg/month, respectively). In the USA alone, neurology (CIDP, MG and some other conditions) is the leading medical field driving the demand for Ig (65-70%) whereas demand for immunodeficiencies (PID and SID) is 25-30%.

New emerging treatments for hematological (such as monoclonal antibodies or CAR T-cells), rheumatological and oncological conditions that may cause antibody deficiency as a side effect have also contributed to a higher demand for short and long-term Ig replacement (11). In the near future, SID (e.g., Chronic Lymphocytic Leukemia) might represent a larger group of immunocompromised patients. Other factors are likely to also be drivers for increased usage of Ig: ageing with higher immune dysregulation conditions in the elderly, higher rates of obesity in the general population, increased indications of long-term immunomodulatory usage of Ig (with higher dosages and personalized dosages), higher number of rare neurological diseases and the impact of emerging pathogens/epidemics/breakthroughs enhanced or not by systemic climate changes — such as Zika virus driven Guillain-Barré syndrome.

Balanced against the increased demands for Ig therapy, novel therapeutic approaches including the targeting of the neonatal Fc receptor, are likely to form part of future treatment strategies for autoantibody-mediated diseases including MG and immune thrombocytopenic purpura (ITP). These treatments would potentially reduce the need for high-dose immunomodulatory doses of Ig (12). In addition, as curative treatments including hematopoietic stem cell transplantation (HSCT) and gene therapy (GT) are used in expanding cohorts of patients with immunodeficiency, a subset of patients will most likely no longer require lifelong IgRT.

However, the supply of Ig is variable and has more recently been impacted by the COVID-19 pandemic (13). Reduced plasma donation rates during the peak of the pandemic, due to lockdowns and social distancing, had a direct impact on supply. Signs of recovery in donation rates in 2021 and 2022 have been seen as donors return to donation centers. This has also been aided by the opening of new donation centers across Europe and the United States. In parts of the world where donation is compensated, the current economic climate is likely to have contributed to increased donations as well. Ig usage had grown at a steady rate of 9% for the past 12+ years (2008–2020) before the pandemic, with a decline of 5-6% during the 2020-2021 period due to plasma collection and Ig supply. North America and Europe encompass most of the consumption of Ig (48% and 25%, respectively) whereas the Asia-Pacific region (mostly China) and the Latin-American continent account for 20% and 3%, respectively.

Currently, Ig is sourced from donations from a limited number of countries globally. In 2019 source or apheresis plasma represented about 90% of the plasma for fractionation collected worldwide, whereas recovered plasma (plasma extracted from blood donations) represented around 10%. North America and four European countries collectively contribute to most of the source plasma collected and therefore immunoglobulin supply significantly relies on plasma collected in the United States. In addition, Ig usage is also highest within these regions. Despite expansion of the Ig market globally, patient access to Ig in many countries is still limited or non-existent. See **Table 1** for the summary on the prospective (Where do we want to be? And how are we going to get there)?.

**Table 1:** The therapeutic evolution of immunoglobulins: alternatives, access and supply.

**Where do we want to be?** • A patient centered care approach is required to ensure immunoglobulins are available and accessible for patients with immune deficiencies for whom there are no alternative treatments. • There is a need for a reliable and diverse supply of immunoglobulins to prevent situations where access to immunoglobulins is jeopardized. • Supply of immunoglobulins needs to be equitable and affordable *via* all healthcare systems for all PID patients of all ages, particularly as demand for immunoglobulin in developing countries increases. • Healthcare systems, driven by an ethical framework, need to provide immunoglobulins for all patients with immunodeficiencies who are eligible for treatment. • Patients with immune deficiencies should have access to personalized choice for immunoglobulin replacement including IVIg, SCIg (including fSCIg, rapid push) and any other novel options.	**How are we going to get there?** • Open communication and collaboration with all stakeholders supplying and using immunoglobulins is required. This may take the form of a consortium and should include multi-specialty representation, patients and commercial stakeholders. These discussions should also include users of alternative treatments for non-PID and SID groups of patients, potentially through regular meetings and activities of the Platform of Plasma Protein Users (PLUS). • Increased plasma collection from more nations across the world is required to improve access globally to immunoglobulin therapies and to prevent over-reliance on limited sources. Plasmapheresis is key to get us there. • In the absence of consensus regarding optimal IgG trough levels in SID patients, global guidance should be developed focusing on immunoglobulins in SID to optimize care. Immunodeficiency organizations including IPOPI and all continental societies for PIDs such as ESID, CID, APSID, ASID, LASID should play a role in the development of evidence based, patient-focused guidance. • In some healthcare systems, demand management systems operate and utilize local and regional immunoglobulin assessment panels and guidelines to govern the use (dose and place of Ig in a given treatment pathway) and reimbursement of Ig. IPOPI, alongside other societies, could replicate some of this guidance to optimize the use of Ig globally and help deliver regionally balanced crisis management. • Regionally balanced plasma collection is required to increase global sufficiency of Ig supply across countries. Re-assessing the role of compensated donations alongside unpaid voluntary donations across the world may be required in addition to exploring the current barriers against donation of blood and plasma and the need to potentially reviewing current donor deferrals which may no longer be justified based on current scientific evidence.

## Part 2: PID personalized management: from diagnosis to treatment

### Molecular diagnosis and newborn screening — where are we now?

There are currently more than 485 inborn errors of immunity (IEI) included in the most recently updated classification from the International Union of Immunological Societies (IUIS) Expert committee published in 2022. This includes 55 more monogenic defects that have been discovered, confirmed or expanded on since the last IUIS update in 2019 (14, 15). Next generation sequencing (NGS) methods such as Whole Exome Sequencing (WES) or Whole Genome Sequencing (WGS) have contributed to the expanding numbers of IEIs identified and have become the standard for both research and clinical diagnostics in some parts of the world. However, access to genetic testing is not uniform and can be very limited, particularly in developing countries.

Depending on the severity of the phenotype, especially in pediatric cases or in multiplex families, NGS (including PID gene panel) can yield results in 30-60% of cases, with increased diagnostic yield using whole genome sequencing (WGS) and RNA sequencing technologies (16, 17). Although in PIDs such as common variable immunodeficiency (CVID) or in adults with unclassified combined ID, this yield can be much lower.

However, identification of novel genetic variants is only the first step, and these variants require validation. The effect of the variant on the gene product, the functional effect of the variant in relevant cell types, and rescue of the phenotype are parameters that help validate the causal relationship between genotype and phenotype (18). Disregarding a variant of uncertain significance (VUS) can be difficult, and at present, there is no clear consensus on who should perform the validation of a genetic result or how this important work should be funded, with clinicians often relying on research groups to assist in clinical validation, which can be a lengthy process largely depending on research priorities. Rapid NGS has been used in critically unwell infants on intensive care unit with direct benefit on clinical care (19).

Another challenge in the field of next generation diagnostics is the absence of a platform for sharing genetic data, while respecting the patient’s privacy. Resolving this could enable collaboration worldwide and help to connect groups working on different genetic variants to share information that will ultimately increase and optimise the diagnosis of PIDs. Also, as population genomic databases collect sequencing data representing mostly north American and European populations, expanding the cohort size of those databases and further diversifying the genomic data to incorporate individuals from underrepresented ancestries will be needed.

### PID Newborn screening- beyond TRECs and KRECs?

Early diagnosis of PIDs reduces irreversible morbidity and mortality and improves quality of life by enabling early initiation of appropriate supportive and definitive treatment. Newborn screening (NBS) has therefore been implemented in certain countries including the United States and several EU countries, among others, to detect T-cell and B-Cell PIDs by the quantification of T-cell receptor excision circles (TRECs) and kappa-deleting-recombination excision circles (KRECs), respectively. In addition to severe combined immune deficiency (SCID), NBS using a combination of TRECs and KRECs can identify other PIDs including 22q deletion syndrome, combined immune deficiencies and X-linked agammaglobulinaemia (20). With the decline in cost and the potential for rapid turnaround time, WGS is likely to be considered for future NBS programs (21). Special consideration should therefore be given to the rapid development of genetic testing to greatly alter the potential for diagnosis at birth, while at the same time ensuring that the ethical challenges are addressed appropriately.

Despite the clear benefits brought about by NBS with asymptomatic detection and access to treatment resulting in significantly improved outcomes, the availability of such programs is variable across the world, most notably in developing countries (22). The importance of working towards equity and innovation in NBS has been highlighted by recent publication of the experience of individual countries within the European Union, initiatives to help countries establish or expand existing NBS programs and development of the European Reference Network (ERN) Expert Platform on Newborn Screening (23, 24). The use of WGS for NBS will likely result in detecting diseases without clear benefit of early detection. This issue of VUS that is increasingly encountered as genomic medicine becomes more prevalent will also impact NBS when WGS methods are used. So, with improved NGS technologies, there is an increased need for more expert centers for the diagnosis and clinical management of PID. See **Table 2** for the summary on the prospective (Where do we want to be? And how are we going to get there)?.

**Table 2:** PID personalized management: from diagnosis to treatment.

**Where do we want to be?** • Widespread accessibility of NGS to facilitate the diagnosis of PID is required. • Improved access to rapid NGS pipelines for critically ill patients where PID is suspected. • Improved availability of appropriate functional tests for VUSs including clear guidance on who is best placed to validate a particular genetic result and fund this work. • Fostering collaborative research efforts through wide data sharing including genetic data. • Introduction and expansion of NBS for additional important PIDs with emphasis on equity of access to NBS programs. • Facilitated implementation of PID NBS programs by calling for mechanisms to share and promote good practices from existing national NBS programs. • More opportunities for scientific training in genetics and interpretation of results. • With improved NGS technologies, there is an increased need for more expert centers for the diagnosis and clinical management of PID. • Efforts are needed to increase awareness of the role of genetic testing among physician dealing with adult patients.	**How are we going to get there?** • Promote data and knowledge sharing and interoperability to improve diagnosis and management of PIDs. • Anonymization of data at the source, regardless of where the data originates, will facilitate data sharing and ensure that the expertise of groups already working on variants or pathways can be accessed and utilized. Working towards a model that centralizes functional tests, with overarching regional reimbursement, or standardization of functional tests across a network would enable robust and time-efficient validation of genetic results. • Broadening of registry consents (e.g., ESID registry consent) to enable samples to be shared will assist in timely validation of genetic results. • Increased awareness of the value of WGS and NBS, notably through participation of PID stakeholders in global awareness raising efforts (i.e., International Neonatal Screening Day). IPOPI, in conjunction with other members of Screen4Rare, continue to work towards improving equity of access and innovation in NBS. • Genetic counselling and future disease prevention in affected families through the expansion of facilities for pre-implantation and prenatal genetic diagnosis is an important component for the future management of PID. • Advances in bioinformatics are required to keep genomic libraries up to date, improve diagnostic accuracy and help to distinguish disease-causing variants from variants of unknown significance.

*ESID, European Society for Immunodeficiencies.

### Targeted and curative treatments

Greater understanding of underlying mechanisms of disease, particularly with the advances in NGS methods and improved gene discovery, has enabled targeted therapies to be developed and used in the treatment of PIDs. Examples include the use of small molecule inhibitors such as JAK inhibitors in STAT1 gain-of-function and STAT3 gain-of-function and biologic immunomodulators such as Abatacept in CTLA-4/LRBA deficiency (25, 26). These targeted treatments reduce the need for non-targeted immunosuppression, such as corticosteroids, that are often associated with many side-effects. This targeted approach can be used as a maintenance therapy or as a bridge to curative treatment with HSCT or gene therapy (27, 28).

Despite these advances in precision medicine, there is often poor and uneven access to new targeted therapies and monoclonal antibodies for PID. Input by expert multidisciplinary teams in PID can also be limited, resulting in reduced access to personalized therapies and early consideration of curative treatments.

In addition to personalized and curative treatments, in some regions patients with PIDs may have limited options and access to anti-viral therapies for treatment of viral infections such as CMV, adenovirus, etc … In part, logistical and funding obstacles create challenges for establishing drug trials in rare disease cohorts, making it difficult for patients with rare disease to access novel treatments. The PID field has not yet fully uncovered and exploited the benefits of accelerated drug development as seen in other specialties such as oncology.

Allogeneic HSCT has been the curative treatment of choice for many severe PIDs presenting in early childhood. With improved patient identification and selection and improvement in HSCT conditioning, the outcomes of HSCT in adults have improved providing a definitive treatment option in some adults with PID (29, 30).

Gene therapy now also provides an alternative curative option for some PIDs with reduced mortality, increasing safety data and no risk of graft versus host disease. However, high-cost personalized products like gene therapy still have a number of logistical and financial challenges to overcome, particularly given the difficulty in commercialization in this area (31, 32). See **Table 3** for the summary on the prospective (Where do we want to be? And how are we going to get there)?.

**Table 3:** Targeted and curative treatments.

**Where do we want to be?** • Universal access to personalized therapeutic approaches for all PID patients, including those living with ultra-rare PIDs. • Access to additional drugs including targeted, (protein-based) immunomodulatory drugs (incl. smart molecules — see point 4 below) and antimicrobials (including antivirals and antifungals) for patients with PIDs. • Commercialization models of gene therapy that are sustainable. • Improved communication with other stakeholders including those in different specialties and within industry to identify potential novel drugs, or drugs that can be repurposed or re-evaluated after initial unsuccessful outcome for a different condition (e.g., oncology). • Innovative clinical trial approaches that take into account the specific challenges of rare diseases such as PIDs.	**How are we going to get there?** • Tackle the growing economic hurdles that are preventing new life-saving gene therapies from getting to the patients who need them the most. (i.e., Consider funding models for gene therapy, e.g., AGORA consortium). • Establish a consortium focused on bringing together public and private parties for drug repurposing and development of new PID targeted therapies. • Replicate other successful approaches and models including those used globally during the SARS-CoV-2 pandemic for PID drug development and implication. • Develop a clinical trial framework for PIDs and explore novel funding channels for clinical trials. There is a need for prospective studies conducted in international consortia.

## Part 3: quality of life of patients with PID - how to evaluate QoL of patients with PID?

With improvements in care of patients with PID, including curative approaches, most outcome data are focused on overall survival and incidence of complications. However, limited data are collected about the impact of these interventions on QoL of patients with PID. The evaluation of QoL is complex and various factors including the most suitable assessment parameters, the tools used in the assessment of QoL in adults *vs.* children, and the impact of cultural differences need to be considered.

Some QoL data in patients with PID are available. The French Reference center for PIDs (CEREDIH) assessed both the health-related quality of life (HR-QoL) of children (33) and adults (34) with PID compared to matched controls using an age-relevant questionnaire. Except for an improved relationship with family and teachers, children and adolescents scored significantly lower in all domains of the QoL assessment, while adults with PID diagnosed in childhood demonstrated a reduction in QoL on all domains including social functioning parameters. A study performed in Spain on pediatric PID patients using a HR-QoL and a multidimensional fatigue questionnaire found a significant increase in fatigue and overall HR-QoL, with emotional distress and work/school related issues being most affected (35). Results from an online QoL assessment provided by IPOPI, with respondents from 21 different countries, confirmed that overall PID patients score below average for physical and mental well-being (36).

A specific QoL score has been developed by the group of Isabella Quinti in Italy (37) which highlights the impact of disease in physical function (e.g. pain, fatigue), emotional (psychological distress, anxiety and depression) and social areas. A six-year prospective follow-up cohort study of 96 Italian CVID patients showed a correlation between low HR-QoL scores in physical and social functioning and an increased risk of anxiety, depression, and death (38). A recent HR-QoL study on CVID using the Patient-Reported Outcomes Measurement Information System (PROMIS)-29 score in the USA for chronic diseases (39–41) emphasizes the relevance to capture the feeling of fatigue as a most salient outcome in CVID patients (42). Similar findings were reported by the national US Immune Deficiency Foundation (IDF) for patients suffering from CVID (43). Moreover, disease adjusted QoL measures are limited and need global validation.

Similarly, most research in patients post-HSCT is centered on physical outcome measures and event-free survival. Data focused on psychosocial outcomes and quality of life post-HSCT are often obtained in children and limited data are available for patients who were treated with HSCT for a PID. Recent data looking at long-term QoL and psychosocial outcomes in adults who underwent HSCT in childhood for PID have been reported and demonstrate mixed outcomes (44). This highlights the need for further research in this area to guide the development of screening protocols and to influence intervention.

Overall, it can be difficult to assess QoL and publish QoL data in PID patients. Current challenges in this area are partly driven by poor engagement with methodologists, hurdles to publish QoL studies in prominent journals, as well as poor availability and engagement of health psychologists.

Patients with PID are central in discussions regarding quality of life and patient involvement in the design of questionnaires, and tools that sensitively collect these data are essential. The importance of patient engagement and the impact that such data may have, including in standardization of disease specific care, should be emphasized. Patient organizations such as IPOPI are well placed to drive and be involved in studies to assess QoL and promote the position of the patient as being the expert. See **Table 4** for the summary on the prospective (Where do we want to be? And how are we going to get there)?.

**Table 4:** Quality of life of patients with PID: How to evaluate QoL of patients with PID?

**Where do we want to be?** • Collection of good quality QoL data using sensitive tools. • Involvement of PID patients in the choice of tools and outcome measures. • Involvement of academic clinical psychologists in research that focuses on collection of psychosocial outcomes and QoL data. • Consideration of broader reasons to collect QoL data and improve the scope of data collected to enable subsequent clinical trial design and improve access to medications. • Validation of disease specific QoL measuring tools	**How are we going to get there?** • Develop an expert taskforce with IPOPI representation to determine the most appropriate QoL tool that is sensitive and practical. Utilization of this tool as part of pilot studies to enable global data collection from patients with PID in different healthcare systems. • Consider a position paper regarding what should be done at national and international level in this area. • Inclusion of QoL data in registries such as the ESID registry. The data output will reflect the time, expertise and funding that is required to collect these data. Therefore, application to international and regional funding bodies to explore funding of QoL data collection and recording should be considered.

## Part 4: the unknown facets of PID- what will the future bring? From PID to IEI

In 2022, the IUIS expert committee on IEI published an updated classification of IEIs, which now includes more than 485 IEIs as defined by single gene defects that underlie a diverse spectrum of phenotypic presentations. These include susceptibility to infection and immune dysregulation (14). Algorithms that include clinical and laboratory parameters help in the classification of these disorders into ten broad categories of IEI. This classification highlights our greater understanding that PID is more than just a susceptibility to infection and emphasizes the non-infectious manifestations of certain IEIs including malignancy, autoinflammation, autoimmunity and allergy. A recent retrospective study of over 1300 patients over a 10-year follow-up period highlighted the severity, as well as the frequency, of non-infectious complications of PID, demonstrating that these disease manifestations are common and no longer an exception (45). In addition, there has been increased understanding that not all PIDs are a result of a deficiency in a protein but can be secondary to ‘overactivation’ in the immune response. Some established PIDs such as chronic granulomatous disease have clear susceptibility to infection as well as granulomatous inflammation. Others are associated with low risk of infection but with higher risk of non-infectious complications, such as autoimmune lymphoproliferative syndrome (ALPS) immune dysregulation, polyendocrinopathy, enteropathy, X-linked syndrome (IPEX).

Over time, there has been evolution of the term PID to IEI among those who work in the field, although the terms are not used interchangeably by all. The term IEI no longer clearly splits conditions into those with susceptibility to infections and those without but encompasses a spectrum of disorders along a continuum. It is evident that there is a lack of clarity regarding terminology and blurring of lines between PID and IEI. Are the terms truly interchangeable or should PID be considered a subcategory of IEIs or vice-versa? The group discussed this aspect, and some consensus was reached that IEIs could be considered a subcategory of PIDs.

In this regard, Primary Immunodeficiencies are considered as conditions presenting with a combination, or not, of autoinflammation, autoimmunity, infection, allergy and malignancy. As inborn and inherited are often considered as identical, we consider that the Inborn Error of Immunity designation should only be used if the molecular (genetic) origin has been identified – which is, to the best of the group’s knowledge, only the case for maximally 50% of PID patients. In addition, as several of the conditions caused by auto-antibodies or somatic mutations have not been attributed to germline mutations, it is difficult to include them in the “inborn” errors, if we consider “inborn” to translate to “heritable”.

The term primary in PID and inborn in IEI may imply that these are pediatric conditions even when many patients are diagnosed with PID/IEIs in adulthood, sometimes without a monogenic cause being found. In addition, not all PIDs may be considered to be inborn and not all PIDs are manifesting in childhood by definition, “e.g. Phenocopies of PID”.

With evolution of terminology, there must be awareness that, from a global patient perspective, it has taken time to increase the awareness of the term PID. The term IEI is not widely used amongst patients and patient organizations. An awareness of this is important as a shift in terminology has implications on patient understanding of their underlying condition.

With the advent of greater treatments for hematological and rheumatological conditions, many immunologists care for many secondary immunodeficiency (SID) patients. The terms PID and IEI do not encompass this growing group of immunodeficiencies and there may be a current tendency for this group of patients to be underrepresented by PID patient groups. Obviously, there is a need to look at this group of conditions and assess whether specific subsets of patients (secondary antibody deficiencies) may benefit from and be relevant to PID patient organizations and their stakeholders. See **Table 5** for the summary on the prospective (Where do we want to be? And how are we going to get there)?.

**Table 5:** The unknown facets of PID: What will the future bring?

**Where do we want to be?** • Use of terminology that encompasses groups of patients that are cared for by immunologists and are represented by patient organizations including the growing group of secondary immunodeficiency patients. • Terminology that provides clarity for patients, parents and carers, clinicians, the multidisciplinary team and researchers. • Understanding that it takes time to increase awareness and knowledge of terminology among patients and clinicians, and the implications of switching terminology need to be considered. • Patients are cared for by different subspecialties that include immunology, internal medicine, rheumatology, hematology, gastroenterology, infectious diseases specialists, allergology, oncology, etc…. There should be more guidelines to help the subspecialty healthcare professionals to care for patients with these specific diseases. • The management of patients with IEI/PID requires a high-level multidisciplinary approach, as we have stated. This is the case in children and teenagers but even more so in adults and the gap between transitioning from the pediatric setting to adult services can be unsettling, due to the heterogeneity of actors in charge.	**How are we going to get there?** • Acknowledgement that any broadening of scope or change in terminology requires gradual transition. • Well defined terminology is paramount to not blur the lines, dissolve messages and risk creating confusion among stakeholders. • In doing so, there is a need to keep in mind the global context and regional specificities/needs. • Utilizing examples of other diseases including rare disease where scope has been broadened or where the chosen terminology encompasses more than what its name points to e.g., World Federation of Haemophilia could act as model.

## Data availability statement

The original contributions presented in the study are included in the article. Further inquiries can be directed to the corresponding author.

## Author contributions

ST, SB, NM, SVC, JP, MP drafted the manuscript. All authors reviewed the manuscript and made significant contributions to the final version. All authors contributed to the article and approved the submitted version.
